# Towards a more precise and individualized assessment of breast cancer risk

**DOI:** 10.18632/aging.101803

**Published:** 2019-02-20

**Authors:** Marie E. Wood, Nicholas H. Farina, Thomas P. Ahern, Melissa E. Cuke, Janet L. Stein, Gary S. Stein, Jane B. Lian

**Affiliations:** 1 University of Vermont Cancer Center, The Robert Larner MD College of Medicine, University of Vermont, Burlington, VT 05405, USA; 2 Division of Hematology and Oncology, The Robert Larner MD College of Medicine, University of Vermont Medical Center, Burlington, VT 05405, USA; 3 Department of Biochemistry, and The Robert Larner MD College of Medicine, University of Vermont, Burlington, VT 05405, USA; 4 Department of Surgery, The Robert Larner MD College of Medicine, University of Vermont, Burlington, VT 05405, USA

**Keywords:** breast cancer risk, precision risk assessment, biomarkers, circulating miRNA

## Abstract

Many clinically based models are available for breast cancer risk assessment; however, these models are not particularly useful at the individual level, despite being designed with that intent. There is, therefore, a significant need for improved, precise individualized risk assessment. In this Research Perspective, we highlight commonly used clinical risk assessment models and recent scientific advances to individualize risk assessment using precision biomarkers. Genome-wide association studies have identified >100 single nucleotide polymorphisms (SNPs) associated with breast cancer risk, and polygenic risk scores (PRS) have been developed by several groups using this information. The ability of a PRS to improve risk assessment is promising; however, validation in both genetically and ethnically diverse populations is needed. Additionally, novel classes of biomarkers, such as microRNAs, may capture clinically relevant information based on epigenetic regulation of gene expression. Our group has recently identified a circulating-microRNA signature predictive of long-term breast cancer in a prospective cohort of high-risk women. While progress has been made, the importance of accurate risk assessment cannot be understated. Precision risk assessment will identify those women at greatest risk of developing breast cancer, thus avoiding overtreatment of women at average risk and identifying the most appropriate candidates for chemoprevention or surgical prevention.

## Breast cancer risk spans a wide range

Individual risk for developing breast cancer varies between 11.6% for women without specific clinical risk factors (i.e., average risk) and 85% for women with pathogenic germline mutations in highly penetrant genes (i.e., *BRCA1*, *BRCA2*, *TP53*, and *PTEN*) [1–6]. Assessment of individual risk is critical for tailoring screening and prevention strategies appropriate to the severity of risk, and therefore avoid unnecessary screening and over-treatment. Women at average risk can delay initiation of screening as recommended by both the United States Preventative Services Task Force and the American Cancer Society [7,8]. Women at moderate risk can begin annual screening earlier and should consider FDA-approved chemoprevention, such as tamoxifen, raloxifene or aromatase inhibitors [9]. Women at highest risk are candidates for aggressive screening (e.g., with breast MRI) or surgical prevention [10–13].

## Limitations of current risk assessment models frequently used in the clinic

A number of models are available for estimation of individual breast cancer risk based on clinical factors such as family history, reproductive profile, history of prior breast biopsy, and breast density (Table 1). The most commonly used clinical models are the Gail [14,15], the Claus [16], and the International Breast Cancer Intervention Study (IBIS) models [17]. For an excellent and comprehensive discussion of all available clinical models (*e.g.*, hereditary, etc.) see the 2017 Cintolo-Gonzalez review [18,19]. The Gail model uses reproductive and biopsy information but only a limited family history (mother or sister with breast cancer) to calculate risk. This model is validated and classifies subsequent breast cancer cases modestly well, with estimates of the area under the receiver-operating characteristic curve (AUC) of 0.45-0.74 [15,20–22]. For risk calculations see https://bcrisktool.cancer.gov. The Claus model uses first- and second-degree family history to calculate risk but does not consider additional family history and other risk factors (such as hormonal factors or biopsy history). This model has an estimated AUC of 0.72 [20]. For risk calculations see CancerGene (https://cagene.com/) [23]. The IBIS model uses reproductive history, biopsy history, family history and body mass index (BMI). The IBIS model also includes a more extensive assessment of family history, characterizing breast cancers in both first- and second-degree relatives and the age at which they were diagnosed. The AUC of the IBIS model ranges between 0.54 – 0.76, depending on the population assessed [20,22,24–28]. For risk calculations see http://www.ems-trials.org/riskevaluator/. See Table 1 for a more complete review of factors included in each model and the discriminatory accuracy in both general and high-risk populations.

**Table 1:** Comparison of commonly used clinical breast cancer risk assessment models: risk factors considered and discriminatory accuracy in independent datasets.

**Model**		**Gail**	**Claus**	**IBIS (Tyrer-Cuzick)**			**BCSC**
**Model version**		**2** ^[^^15^^,^^105^^–^^108^^]^	**1** ^[^^16^^,^^109^^]^	**6.0.0** ^[^^17^^]^	**7.0.2**	**8.0**	**2.0** ^[^^40^^]^
	**Personal**						
	Age	X^a^	X	X	X	X	X^a^
	BMI			X	X	X	
	Race/ethnicity	X		X	X	X	X
	**Hormonal**						
	Age at menarche	X		X	X	X	
	Menopausal status			X	X	X	
	Parity, age first birth	X		X	X	X	
	HRT use			X	X	X	
	**Benign Breast Disease (BBD)**						
	Num. breast biopsies	X					
	BBD with LCIS			X	X	X	X
	BBD with atypia	X		X	X	X	X
	BBD without atypia			X	X	X	X
	**Family history**						
	1° female relatives (breast)	X^b^	X	X	X	X	X^b^
	Extended family hx (breast)		X	X^c^	X^c^	X^c^	
	1° male family hx (breast)				X	X	
	Family hx of ovarian cancer			X	X	X	
	**Genetic variants**						
	BRCA status			X	X	X	
	Polygenic Risk Score (PRS)					X	
	**Breast density**					X	X
**Breast cancer outcomes**		Invasive	Invasive + DCIS	Invasive + DCIS			Invasive
	5-yr risk	X	X		X	X	X
	> 10-yr risk^d^	X	X	X	X	X	X
**General population (AUC)**		0.54-0.67 ^[^^26^^,^^27^^,^^106^^–^^108^^,^^110^^–^^116^^]^		0.57-0.695 ^[^^26^^,^^27^^]^			0.66 ^[^^50^^]^
**High-risk women (AUC)**		0.45-0.735 ^[^^20^^,^^22^^]^	0.716 ^[^^20^^]^	0.51-0.762 ^[^^20^^,^^22^^,^^28^^]^	0.54 ^[^^24^^]^		

a Model not applicable for women under age 35.

b Ages of diagnoses not considered.

c 1° and 2° female relatives, as well as selected 3° relatives (female first cousins), diagnosed with breast cancer.

d Risk of developing breast cancer outcome by age 90 (Gail model); by age 79 (Claus model); within 10 years and by age 80 (IBIS model 6); to age 85 (IBIS models 7 and 8), and over a 10-yr age interval (BCSC model).

Newer clinical models such as the Breast Cancer Surveillance Consortium (BCSC) model and updated/revised versions of the IBIS model (version 8) have incorporated mammographic density (MD) into assessment of risk. Mammographic density is a strong, independent risk factor for breast cancer development with studies showing a 4-6-fold increased risk for breast cancer for women with the highest breast density category compared with women in the lowest breast density category [29–38]. The BCSC model also incorporates reproductive factors, first-degree family-history, and recently added biopsy history to its set of predictors [39,40]. This model is validated and classifies breast cancer incidence with an AUC of 0.67 [39,41]. Accuracy of the latest version of the IBIS model has not been assessed.

Given that an AUC of 0.5 suggests that the test (or model in this case) performs no better than chance, the fact that none of the above models have an AUC greater than 0.76 leaves room for improvement [22,42,43]. There is, therefore, a significant need for more precise risk assessment. Recent advances in genetics have improved our ability to assess risk at the individual level. Genome-wide association studies have identified >100 single nucleotide polymorphisms (SNPs) associated with breast cancer risk [44–47] and polygenic risk scores (PRS) have been developed by several groups using this information [48,49]. Case-control studies have demonstrated the ability of PRS to accurately categorize risk (with AUC ranging from 0.59 – 0.65) [50–52]. However, risk associated with any of the developed polygenic risk scores needs to be interpreted with caution as their predictive capacity has not been validated outside of the populations in which they were developed. As seen with genetic testing, this may limit generalizability [53]. Several groups have examined whether use of PRS improves accuracy of currently available clinical models and demonstrated AUCs between 0.62 and 0.70 [41,54–59]. The ability of PRS to improve current clinical models is under prospective evaluation in the WISDOM trial [60,61]. Given that many of the SNPs included in polygenic risk scores are likely associated with hereditary risk, caution should be used when adding genetic factors to family history-based models without accounting for joint influences on model fit. The ability of a PRS to improve risk assessment is promising; however, utility in genetically and ethnically diverse populations must be studied.

## Use of circulating miRNA biomarkers augment clinical tools to provide personalized risk assessment

Novel classes of biomarkers, such as circulating microRNA (C-miRNA) have emerged as promising cancer biomarkers [62–65] and may provide additional risk information. MicroRNAs (miRNA) are short, non-coding RNAs that bind to target mRNA and inhibit protein expression to regulate cellular processes such as proliferation, differentiation, and apoptosis [66–68]. A single miRNA can simultaneously target hundreds of genes, acting as a master regulator of entire biological pathways, with established roles in controlling normal development and tissue homeostasis [69–71]. Aberrant expression of miRNAs has been shown to regulate cancer cell activity by modulating oncogenic or tumor suppressor pathways to promote disease onset and progression [70]. In addition, miRNAs circulate, acting as intercellular signaling molecules, and may function to establish local and systemic environments for initiation and/or progression of cancer. Circulating miRNAs (C-miRNAs) are released from almost all cells in a variety of forms: in microvesicles [72], exosomes [73], bound to protein or lipid particles [74,75] or as free species [69]. Importantly, miRNAs are readily detectable, stable in circulation and found in most body fluids (e.g. blood, urine) [62,76], all characteristics of an ideal biomarker. The importance of standardized analysis of C-miRNA has become increasingly recognized by our group and others as essential for generating reproducible and actionable results [77–79].

In breast cancer patients, the presence of miRNA in circulation correlates with expression of that miRNA in primary breast tumors [80–82]. Additionally, significant differences in specific C-miRNA have been found between cancer patients and healthy controls [65,83–86], suggesting potential clinical utility for cancer detection [64,81,82,87–93]. For cancer risk assessment a biomarker must predict disease status with acceptable specificity and sensitivity [94]. To date, only a handful of studies have evaluated the utility of C-miRNA in cancer risk assessment. For example, several studies have evaluated miRNAs associated with risk for colon cancer and identified miRNAs associated with a pre-neoplastic colon lesion [95–99]. An independent and larger study identified a panel of 3 C-miRNAs as a promising colon cancer risk biomarker [100]. Other studies have discovered a number of miRNAs dysregulated in women <18 months from a breast cancer diagnosis, consistent with early detection [101–103]. Taken together, these data suggest that it is feasible that C-miRNAs can provide a signature of breast cancer risk with actionable lead-time for prevention.

**Figure 1:**
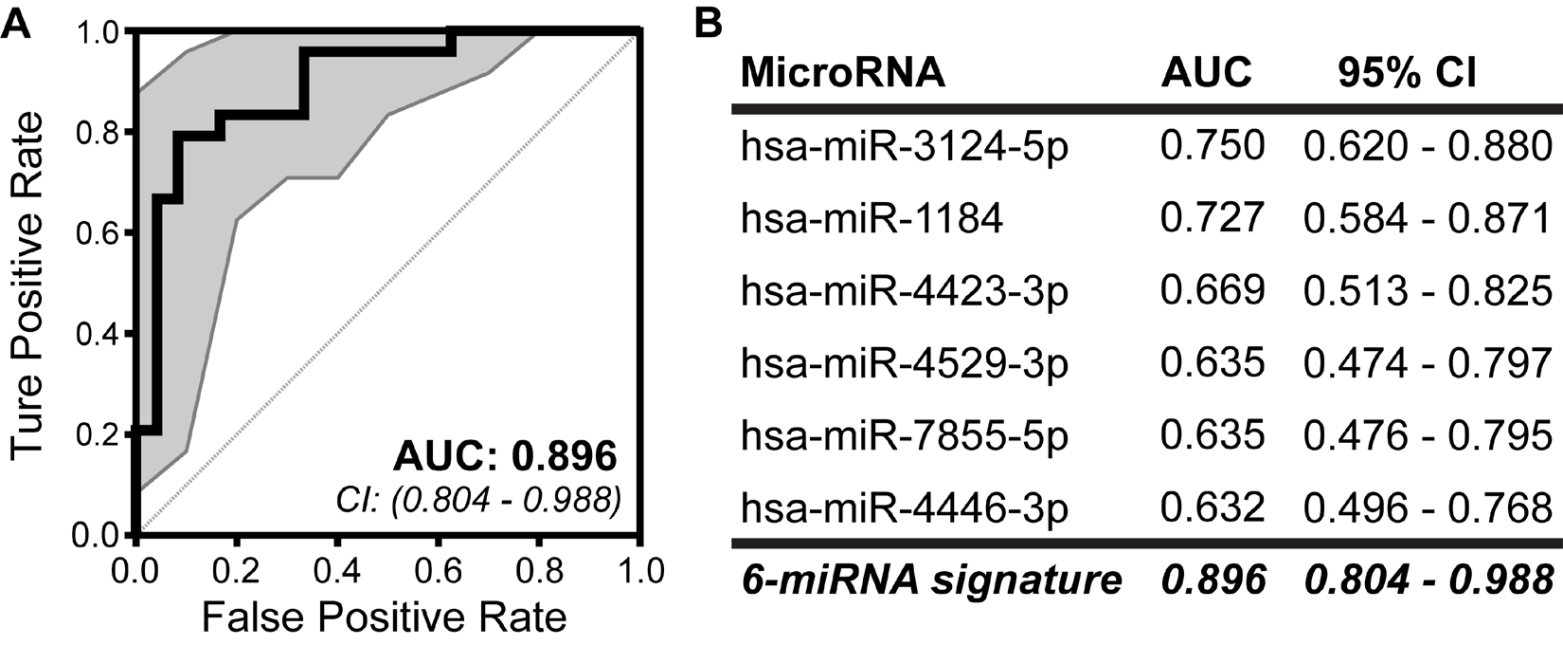
**Development of a predictive miRNA signature for breast cancer risk among high-risk women.** The predictive ability of A) the 6-miRNA risk signature and B) each individual C-miRNA was assessed by ROC curve and AUC based on calculated risk score. The combined expression of the 6 C-miRNAs discriminate cases from controls with increased accuracy and precision than any single miRNA. 95% confidence intervals (CI) are indicated by gray area around each curve. Modified from our 2017 Oncotarget publication [104].

Our group recently identified a C-miRNA-based risk signature predictive of long-term risk in a prospective cohort of women at increased risk for developing breast cancer. This IRB-approved prospective cohort includes over 600 high-risk women (who have signed informed consent) with a median follow-up of 8.9 years. From this cohort we selected 24 invasive breast cancer cases, to whom we matched controls on age, reason for high-risk status (*e.g.*, strong family history of breast cancer or benign breast disease), and follow-up time. The median age at blood draw was 55.4 (range 33.9-77.5) for affected cases and 55.1 (range 32.8-78.4) for cancer-free controls (see Table 1: Subject characteristics in Oncotarget [104] for complete cohort clinical characteristics). RNA was isolated from banked serum, and profiled for over 2500 mature human miRNAs. The full Affymetrix GeneChip miRNA v4 (miRbase v20) microarray expression dataset is freely available in GEO Datasets (GSE98181, https://www.ncbi.nlm.nih.gov/geo/query/acc.cgi?acc=GSE98181) and the R scripts used for data analysis accompany our open access 2017 Oncotarget manuscript as a supplement [104]. We identified 25 C-miRNAs that were significantly differentially expressed between cases and controls. From these 25 miRNAs, we discovered a group of 6 C-miRNAs that together discriminated cases from non-cases with high accuracy (AUC=0.896) (Figure 1). For the women who developed cancer in this cohort, blood had been banked a median of 3.2 years (range 0.6-8.7) prior to diagnosis, making this clearly a signature associated with risk and not early detection [104]. Refinement and validation of this risk signature is ongoing, using banked samples from previously performed randomized clinical trials. The validation of a sensitive and specific, non-invasive C-miRNA risk assessment tool will arm clinicians with vastly improved individualized risk estimates for patients, relevant to both young and older women. These risk estimates can be used to guide selection of the most appropriate screening and prevention options for a given individual. Information from miRNA expression will also provide valuable insight into the underlying biology of breast cancer initiation and may provide targets for chemoprevention.

Personalized and precise risk assessment can identify those women at greatest risk to develop breast cancer, thus avoiding overtreatment of women at lower/average risk and identifying women at high risk who would be candidates for high risk screening, chemoprevention or surgical prevention. Progress has been made towards personalized risk assessment and some promising new markers have been identified. However, rigorous validation of the most promising markers, and the predictive models they contribute to, in relevant populations is necessary before deployment for clinical use.
